# Robot-aided in vitro measurement of patellar stability with consideration to the influence of muscle loading

**DOI:** 10.1186/s12938-015-0068-7

**Published:** 2015-07-23

**Authors:** Andrea Lorenz, Evgenij Bobrowitsch, Markus Wünschel, Christian Walter, Nikolaus Wülker, Ulf G Leichtle

**Affiliations:** Department of Orthopaedic Surgery, University Hospital Tübingen, Hoppe-Seyler-Straße 3, 72076 Tübingen, Germany

**Keywords:** Patellar stability, Patellar displacement, Muscle loading, In vitro testing, Robot-aided measurement

## Abstract

**Background:**

Anterior knee pain is often associated with patellar maltracking and instability. However, objective measurement of patellar stability under clinical and experimental conditions is difficult, and muscular activity influences the results. In the present study, a new experimental setting for in vitro measurement of patellar stability was developed and the mediolateral force–displacement behavior of the native knee analyzed with special emphasis on patellar tilt and muscle loading.

**Methods:**

In the new experimental setup, two established testing methods were combined: an upright knee simulator for positioning and loading of the knee specimens, and an industry robot for mediolateral patellar displacement. A minimally invasive coupling and force control mechanism enabled unconstrained motion of the patella as well as measurement of patellar motion in all six degrees of freedom via an external ultrasonic motion-tracking system. Lateral and medial patellar displacement were measured on seven fresh-frozen human knee specimens in six flexion angles with varying muscle force levels, muscle force distributions, and displacement forces.

**Results:**

Substantial repeatability was achieved for patellar shift (ICC(3,1) = 0.67) and tilt (ICC(3,1) = 0.75). Patellar lateral and medial shift decreased slightly with increasing flexion angle. Additional measurement of patellar tilt provided interesting insights into the different displacement mechanisms in lateral and medial directions. For lateral displacement, the patella tilted in the same (lateral) direction, and tilted in the opposite direction (again laterally) for medial displacement. With regard to asymmetric muscle loading, a significant influence (p < 0.03, up to 5 mm shift and 8° tilt) was found for lateral displacement and a reasonable relationship between muscle and patellar force, whereas no effect was visible in the medial direction.

**Conclusion:**

The developed experimental setup delivered reproducible results and was found to be an excellent testing method for the in vitro analysis of patellar stability and future investigation of surgical techniques for patellar stabilization and total knee arthroplasty. We demonstrated a significant influence of asymmetric quadriceps loading on patellar stability. In particular, increased force application on the vastus lateralis muscle led to a clear increase of lateral patellar displacement.

## Background

Anterior knee pain is a common problem, especially in the young, active population [1–3], but also after surgical interventions such as total knee arthroplasty [4–6]. In addition to patients with non-specific anterior knee pain, multifactorial causes of anterior knee pain are known, including damage or deformities to patella cartilage, limb malalignment, muscular imbalances, and patellar instability. The latter is often associated with anterior knee pain, but can also be a problem without anterior knee pain. Reasons for patellar instability can be divided into osseous abnormalities (e.g. patella alta, limb malalignment or trochlear dysplasia) and soft-tissue abnormalities of the passive (e.g. insufficient capsule and retinacula) or active (e.g. muscular imbalances) structures [7, 8]. Correction of osseous abnormalities or passive soft tissue is difficult and can be managed only surgically, but muscular imbalances can be influenced by muscle training. Therefore, knowledge of the stabilizing ability, especially of the quadriceps muscle, is of great interest.

For testing the stabilizing effect of the muscles, (1) a measure for patellar stability must be defined and (2) an appropriate measurement device must be developed. From a technical viewpoint, patellar stability can be defined as the ability of the patella to return to the stable equilibrium position—i.e. the middle of the femoral trochlear groove—after perturbation [9]. In contrast, orthopedic surgeons use the term “patellar instability” for repeated subluxation or luxation of the patella caused by increased mediolateral shift or tilt of the patella during daily activities. To take into account force–displacement behavior and to preserve certain comparability with clinical methods, “patellar stability” was defined as mediolateral displacement of the patella during controlled application of force in the present study.

In clinical studies, measurement of patellar stability is of considerable interest with regard to non-operative (e.g. muscle training) or operative (e.g. lateral release, reconstruction of the medial patellofemoral ligament) interventions to improve patellar stability, but also to analyze the relationship between anterior knee pain and knee function. In addition to the clinically applied apprehension test and manual testing of patellar mediolateral displacement, a traditional way to quantify the success of a patellar stabilizing treatment is measurement of geometric parameters from radiographs and magnetic resonance images (e.g. static mediolateral patellar shift and tilt) [10–12]. This method is enhanced by applying an external, mediolateral force onto the patella during recording of the radiograph [11, 13, 14]. Other studies involve use of self-developed mechanical or electrical devices for the measurement of patellar shift during mediolateral force application without the need for radiographs [15–17]. These in vivo methods consider the situation without active muscle loading.

For systematic examination of the muscular influence on patellar stability, in vitro experiments are needed. Biomechanical in vitro studies focusing on patellar stability have considered one [18], three [19–21] or five [9, 22–25] parts of the quadriceps muscle and applied forces between 20 N [9] and up to 600 N [20]. However, in many of these studies, the conclusions have been drawn based on kinematics or pressure measurements and are, therefore, associated more with patellar maltracking [18–21, 26]. Only a few studies have measured patellar stability—in this case defined as the force needed for a specified mediolateral displacement of the patella [9, 22–25]. All these studies have used a similar testing device based on a material testing machine (Instron), which was established by Fahramand et al. [9] and improved by Senavongse et al. [25]. In this experimental setting, loading was applied to five parts of the quadriceps muscle by cables routed via pulleys to hanging weights. The total weight (175 N in most studies) was applied to the muscle parts according to their directions and physiologic cross-sectional areas (PCSA) determined in a previous study. Although this apparatus is well established, there remain some potential opportunities for improvement: an upright, more physiologic testing position; less invasive coupling of the displacement device to the patella; or the possibility for additional measurement of patellar tilt during displacement.

For this reason, a new testing rig should be established for advanced testing of mediolateral patellar stability addressing these potential improvements. Based on an adjusted upright knee simulator with adaptable muscle forces for the mounting and loading of specimens, the patella should be displaced minimally invasively using an industry robot, and patellar tracking measurement should be undertaken in all six degrees of freedom. The first aim of this study was to demonstrate the reproducibility of the new setting and determine reasonable testing parameters (muscle force level, muscle force distribution, patellar displacement force, flexion angle). The second aim was to evaluate the mediolateral force–displacement behavior of the patella in the native knee with special regard to the coupling of patellar shift and tilt and the influence of asymmetric muscle loading to provide further insights into how far specific muscle training can influence patellar stability.

## Methods

**Figure 1:**
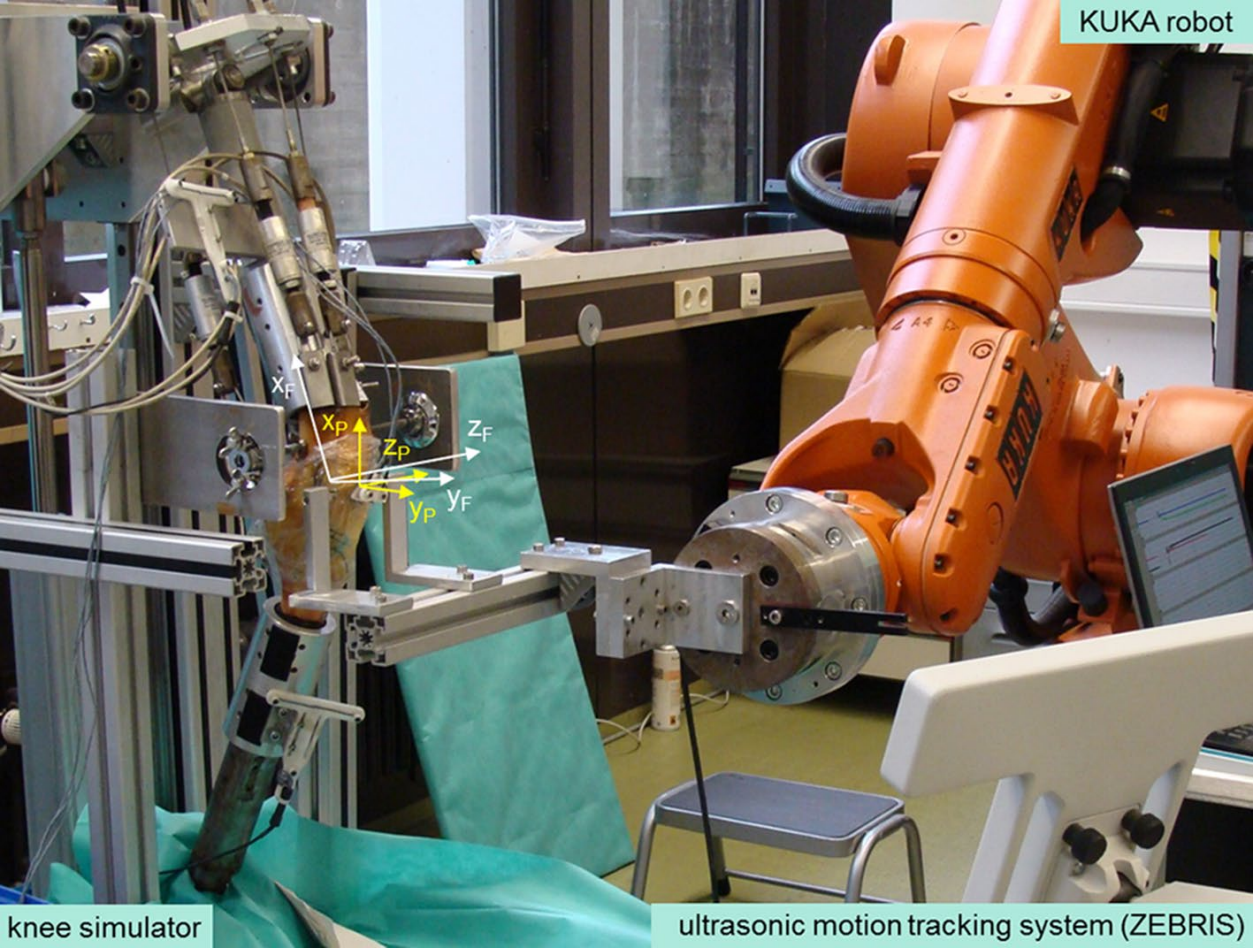
Overview of the experimental setup. Arrangement of the knee simulator, KUKA robot, and ultrasonic motion tracking system (ZEBRIS) during execution of the experiments. The coordinate systems of the femur (x_F_, y_F_, z_F_) and patella (x_P_, y_P_, z_P_) are illustrated. Patellar shift is defined as translation of the patella along the mediolateral axis of the femur (z_F_) and patellar tilt is defined as rotation of the patella around its proximodistal axis (x_P_).

An experimental procedure for the measurement of patellar stability was developed combining two established testing machines at our biomechanical laboratory (Figure 1). For the positioning and muscular loading of the knee joint, a modified knee simulator was used [20, 27, 28]. Displacement of the patella was undertaken by a KUKA industrial robot [29, 30].

**Figure 2:**
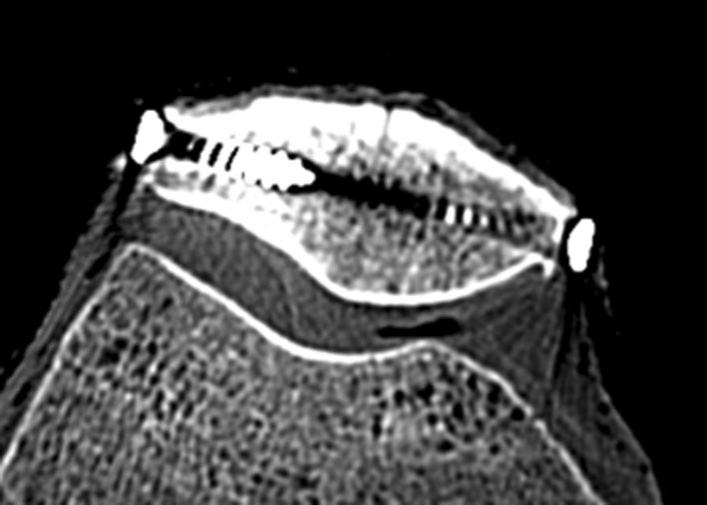
Positioning of patellar screws. We ensured that screws were positioned in one line, with their tips pointing together and to the center of the patella. The CT scan shows a transverse section of a typical patella at the screw level.

Seven fresh-frozen human knee specimens were studied. The stability of the capsule and ligament was examined clinically and additional radiographs and CT scans were undertaken. Knees with severe degeneration, and deformities such as trochlear dysplasia or an insufficient joint capsule, were excluded. Prior to the measurements, specimens were thawed overnight at room temperature. The skin and other soft tissue was removed, preserving the tendons of five major muscles [m. vastus medialis (VM), m. rectus femoris (RF), m. vastus lateralis (VL), m. biceps femoris, m. semimembranosus]. The joint capsule, retinacula and ligaments were kept intact and the fibula screwed to the tibia. For the definition of reproducible coordinate systems and attachment of the robot to the patella, the most prominent points at the femoral epicondyles and in the transversal direction of the patella and tibia were marked with torx screws. Patella screws were also used as the points of force application, so attention was paid that they were positioned in one line with their tips pointing together and to the center of the patella (Figure 2). The femur and tibia were cut 15 cm from the joint line. Their ends were embedded into aluminum cylinders using bone cement and fixed to the respective modules at the knee simulator, paying attention that the posterior femoral condyles were positioned parallel to the testing rig. The five dissected muscle tendons were connected to servo motors [28]. The knee simulator was used for upright positioning of the knee joint in different flexion angles (15°, 30°, 45°, 60°, 75° and 90°) and for loading of the muscles. Each of the hamstring muscles was loaded with 20 N. For the quadriceps muscle, the total force (30, 150, 300 or 600 N) and the force distribution were varied: (1) central, symmetric loading (33% at each of the quadriceps parts), (2) mainly lateral loading (67% VL, 33% RM, 0% VM) and (3) mainly medial loading (0% VL, 33% RF, 67% VM). Positioning and loading of the knee were carried out according to previous kinematic measurements [20, 27, 28, 31].

**Figure 3:**
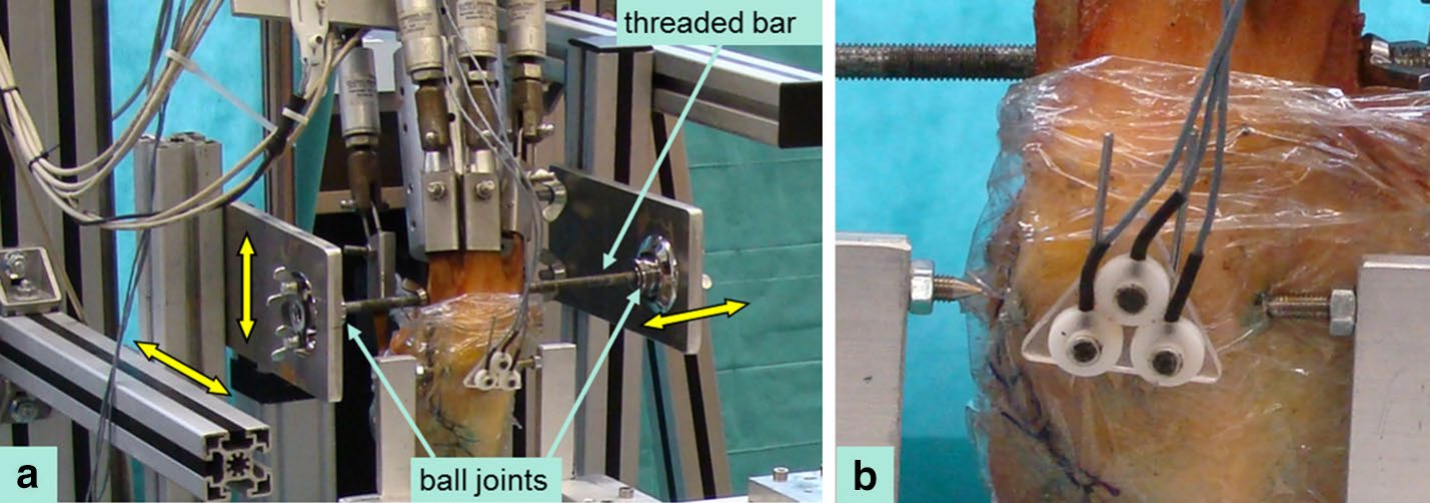
Details of the experimental setup. **a** Mechanism for fixation of the femur to the testing rig in an arbitrary position. A threaded bar was screwed through the femoral shaft and secured to a multiple adjustable frame (*yellow double-arrows* indicate possibilities for adjustment) using two ball joints. The tibia was not constrained further. **b** Robotic patellar attachment via a tip into a torx screw. The two tips are for medial and lateral displacement, respectively, but only one tip is touching the corresponding patellar screw at a time, thereby allowing unconstrained patellar rotation.

The knee simulator enabled knee-joint motion in all six degrees of freedom, so an externally applied force onto the patella would generate motion of the whole knee specimen. To prevent such undesired motion, an additional mechanism for the fixation of the femur to the testing rig was constructed using a threaded bar, ball joints, and a multiple adjustable frame (Figure 3a). Motion of the tibia was not constrained further.

For force application and displacement of the patella, a robotic system with six degrees of freedom (KUKA KR 60-3 robot, Augsburg, Germany; reproducibility: ±0.06 mm) including a universal force/torque sensor (ATI UFS: Theta SI1000-120; resolution: 0.25 N and 0.025 Nm) was used. To allow unconstrained motion of the patella, the robot contacted the patella via a tip, engaging into the torx screw at either the medial or the lateral side of the patella (Figure 3b). For each measurement, the two tips at the end of the robot arm were aligned with the two torx screws in the patella, but only one screw actually contacted the patella at a time. The robot applied different forces in the mediolateral direction (50, 100 N) and simultaneously controlled the forces in anteroposterior and proximodistal directions to be zero, adjusting for the position. No rotation of the robot was applied via the tip, thereby enabling free rotation of the patella. The final position of each measurement was held for 2 s to guarantee a steady state. For each condition and specimen, the patella was displaced three consecutive times in lateral and medial directions. To prevent dehydration of specimens, they were moistened regularly with saline solution and wrapped loosely into thin plastic film during measurements.

The robot was not fixed rigidly to the patella, so it could not be used for measurement of patellar displacement in all six degrees of freedom. Therefore, translations and rotations of the patella were measured using an ultrasonic motion capture system (Zebris CMS-HS, Isny, Germany; resolution: 0.085 mm, accuracy: 1 mm) with a sampling rate of 10 Hz. The kinematics measurement was started 2 s prior to the robot actuator to determine the initial positions of patella and femur (mean value over the first 20 data points). The definition of coordinate systems and calculation of the relative motion of the patella with respect to the femur were undertaken according to the methods described in [20]. Only the patellar tilt was defined in the reverse direction in accordance with the mediolateral patellar shift (lateral shift/tilt: positive values, medial shift/tilt: negative values, Figure 1).

Data analysis was carried out using MATLAB. The three consecutive displacement cycles were separated based on algorithms from [32] and filtered with a moving-average filter over 20 samples (2 s). The maximum mediolateral shift of the smoothed signal was determined for each of the three cycles and averaged. Patellar motion in the other directions was determined at the time of the maximal shift. All variables were set to zero in the initial position. Thus, the displacement generated by the robot was analyzed, not the relative position of the two bones after force application. A total of 2,016 measurements were carried out; 10 data files were erroneous and had to be excluded and 33 files had to be analyzed manually due to problems with the automatic cycle detection. Patellar mediolateral shift and tilt were found to be the most interesting displacement parameters. The effect of asymmetric muscle loading was analyzed statistically using the Friedman test as a non-parametric method for repeated measures.

To demonstrate reproducibility, five repeated measurements in 15° and 60° of flexion at different force levels were executed with one specimen. Furthermore, at the beginning of each test day, the same test condition (15° of flexion, 150 N equally distributed quadriceps force, 50 N patellar force, lateral displacement) was repeated with each specimen (3–4 test days/specimen). Reproducibility was analyzed using the intraclass correlation coefficient (ICC). The two-way mixed model ICC(3,1) was used because all experiments were (and will be) carried out by our robot, and during the measurement series only one repetition was evaluated. To demonstrate the repeatability of the three consecutive measurements of the robot, which are averaged for all measurements, ICC(3,k) was used. For interpretation of ICC agreement values, Landis criteria [33] were used. We aimed for substantial (ICC = 0.61–0.80) to almost perfect (ICC = 0.81–1.00) reliability.

## Results

The three consecutive measurements carried out for each condition showed almost perfect repeatability. The standard deviation was below 0.5 mm and 0.5° for almost all conditions and specimens and an ICC(3,k) > 0.99 was computed for both shift and tilt, considering all measurements. For the reproducibility tests with one specimen and different conditions, the standard deviation was below 0.9 mm and 0.6° and again, almost perfect ICC(3,1) > 0.99 was reached. The repeated measurements of the specified 15° condition in the morning of each test day resulted in standard deviations below 1.5 mm for patellar mediolateral shift. Considering the measurements of the first 3 test days (which were available for all seven specimens), substantial repeatability was achieved with ICC(3,1) = 0.67 and ICC(3,1) = 0.75 for patellar shift and tilt, respectively. A tendency towards an increasing or decreasing displacement over the duration of the tests was not found for any specimen.

**Figure 4:**
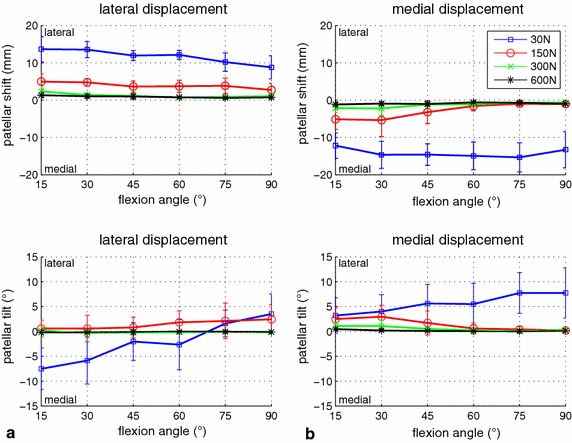
Influence of the quadriceps force level on patellar displacement. Patellar mediolateral shift (*top*) and tilt (*bottom*) during lateral (**a**) and medial (**b**) force application are illustrated, comparing the four measured levels of quadriceps force (30, 150, 300, 600 N). Mean values and standard deviations are shown for 50 N of mediolateral patellar displacement force and central quadriceps force distribution.

With regard to symmetric force distribution, different force levels led to large variations of patellar shift between 1 mm (for 600 N quadriceps load) and 15 mm (for 30 N quadriceps load). Patellar shift decreased with increasing flexion angles for both lateral and medial displacement, except for medial displacement and a 30 N quadriceps load (Figure 4). For lateral shift, the effect of the force level was similar for all flexion angles, and some kind of threshold was found for medial shift. For medial displacement, larger medial shift was coupled with increased lateral tilt. In contrast, for lateral displacement, lateral tilt increased with the flexion angle (i.e. it increased with decreasing lateral shift). Except for the combination of small flexion angles with 30 N quadriceps force and lateral displacement, the patella always tilted laterally (Figure 4).

**Figure 5:**
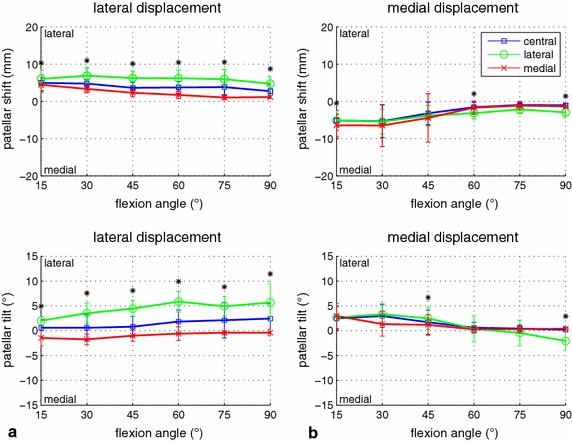
Influence of asymmetric quadriceps loading on patellar displacement for moderate muscle force. Patellar mediolateral shift (*top*) and tilt (*bottom*) during lateral (**a**) and medial (**b**) force application are illustrated, comparing the influence of three quadriceps force distributions (central, lateral, medial). Mean values and standard deviations are shown for 50 N mediolateral displacement force and a total of 150 N quadriceps force. Significant differences are identified by an *asterisk*.

**Figure 6:**
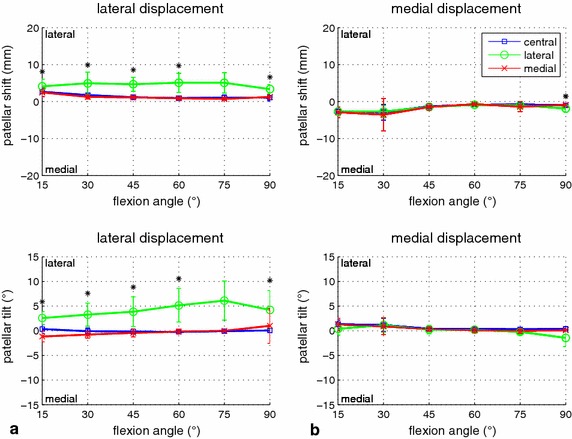
Influence of asymmetric quadriceps loading on patellar displacement for high muscle force. Patellar mediolateral shift (*top*) and tilt (*bottom*) during lateral (**a**) and medial (**b**) force application are illustrated, comparing the influence of three quadriceps force distributions (central, lateral, medial). Mean values and standard deviations are shown for 50 N mediolateral displacement force and a total of 150 N quadriceps force. Significant differences are identified by an *asterisk*.

With regard to asymmetric loading of the quadriceps muscle, for lateral displacement, the influence depended on the configuration of muscle force (MF) and patellar force (PF). At a moderate force relationship (150 N MF and 50 N PF, 300 N MF and 100 N PF), the muscle force distribution had a significant influence on patellar shift and tilt (p < 0.03 for at least five of the six evaluated flexion angles, Figure 5). Differences of up to 5 mm shift and 8° tilt were found between mainly medial and lateral muscle loading. Mainly lateral muscle loading led to more lateral shift and tilt, and mainly medial muscle loading led to more medial shift and tilt. For increasing muscle forces (300 N MF and 50 N PF, 600 N MF and 100 N MF), the effect of mainly medial loading disappeared (difference between central and medial loading <1 mm/°). However, the influence of mainly lateral loading was even larger (Figure 6), and the remaining differences of up to 4 mm shift and 7° tilt were still statistically significant for five of the six flexion angles (p < 0.05). In contrast, for comparably small muscle forces (30 N MF; 150 N MF and 100 N PF), and for the largest measured force difference (600 N MF and 50 N PF) as well as for medial displacement and all measured force configurations (Figures 5, 6), the effect on patellar shift was below 3 mm and statistical significances were found only for a few sparse conditions.

## Discussion

In the present study, a new experimental setting for in vitro measurement of patellar stability was developed and appropriate testing parameters were determined. The experimental setting (which is based on an established knee simulator for kinematics measurements [20, 27, 28]) enables measurement of patellar displacement during mediolateral force application in all six degrees of freedom. Substantial reproducibility of the experiments was demonstrated using the ICC. Also, the independency of the results from the duration of the measurements over several days was shown.

Considering patellar displacement in relation to the flexion angle, slightly decreasing patellar shift was measured for increasing knee flexion. This is attributed to the fact that the patellofemoral contact force increases with the flexion angle [34]. Our results confirm the findings of previous studies [23, 25], whereby between 20° and 90° of flexion a higher displacement force was measured at higher flexion angles to reach a specified mediolateral shift.

In our study, the mediolateral tilt of the patella could also be measured, thereby providing interesting findings. The patella was mostly tilting laterally for both lateral and medial displacement, so different displacement mechanisms for the two directions might have been present. With regard to lateral displacement, the lateral patellar facet was touching the lateral condyle. Subsequently, the displacement force must have generated a lateral torque because a lateral tilt was moving along with the lateral shift. The increasing lateral tilt for higher flexion angles might be explained by the increasing depth of the trochlear groove [35]. With respect to medial displacement, the medial patellar facet was touching the medial condyle. However, in this case, the patella was tilting laterally again—and thus against the displacement direction. This phenomenon might be explained by the shape of the medial patellar facet, which mostly is shorter and steeper than the lateral facet [36], thereby leading to higher contact and friction forces. An additional reason might be the passive stabilizers, which seem to be tighter at the lateral side than at the medial side. In any case, the patella could start climbing the medial condyle only when it is tilted laterally at the same time. This mechanism could explain the strong relationship between shift and tilt as well as the threshold found for medial displacement. For higher flexion angles, the groove is deeper and the contact force higher, so it becomes more difficult for the patella to climb up the condyle. However, when interpreting our data concerning patella tilt, the method used for force application in our experiments should be taken into account, since this might also have some influence on the results.

There were only a few conditions for which medial patellar tilt was measured (e.g. for small muscle forces, small flexion angles and lateral displacement) (Figure 4). These small muscle loadings were applied to keep the knee extension mechanism upright and should demonstrate the unloaded case. Therefore, the results mainly reflect the influence of the passive stabilization structures (joint capsule and retinacula). The effect of muscle loading is only marginal and the influence of the bone surface geometries is also considered small because the patellofemoral contact force is small.

In addition to this low muscle force level (10 N at each of the three considered quadriceps parts), three further force levels were considered (50, 100 and 200 N at each muscle). These force levels were chosen to cover the range of quadriceps forces during squatting experiments with our knee simulator, whereby the total quadriceps force was increasing continuously until approximately 600 N at 90° of flexion [20, 27]. Although this system might not reach a physiologic level, higher quadriceps forces could not be simulated due to the risk of tendon rupture [28]. However, our experiments showed that a total quadriceps force of 150 N yielded the most interesting results because at higher force levels almost no displacement occurred and at a smaller force level the effect of asymmetric muscle loading disappeared. This finding corresponds to previous studies, whereby a comparable force level (175 N) was simulated [9, 24, 25]. With regard to the considered quadriceps parts and distribution of forces, the direction of the overall resultant force is important. For symmetric loading, the resultant force was pointing in the direction of the RF (0°) in our experiments. Compared with other studies, in which the quadriceps was split into five parts and the forces where chosen according to PCSA, resultant forces of 1.1° in the lateral direction (with respect to the RF, Fahramand et al. [37]), 3.1° in lateral [38] and 3.7° in medial directions [21] were calculated. With respect to asymmetric loading, the variation between different studies was even greater, and the direction of the resultant force of mainly lateral loading in our experiments was within the range of the literature for missing vastus medialis obliquus (VMO) [21, 37, 38].

The different displacement mechanisms in lateral and medial directions indicate that enabling of the rotational degrees of freedom, as in our experimental setting, is essential. In this regard, our technique of external force application by a robot via a metal tip that engages into screws at the side of the patella has a similar effect as the ball joint used in [25]. With both techniques, the force is applied in the mediolateral direction, approximately 10 mm posterior to the ventral patellar surface. In the other directions, no forces or torques are applied and the natural mobility of the patella is conserved. However, some additional patellar tilt can be induced, if the instantaneous center of rotation is not located on the action line of the applied force in the transversal plane. This action line might be slightly different in the two settings, as the direction of fore application was aligned with the femur in the study of Senavongse et al. [25] and with the patella in our study. Our technique is less invasive, so it might be easier to apply and the risk of patellar fracture may be smaller (especially after total knee arthroplasty with patellar resurfacing). The screws used in our experiments did not impact on the ability of the patellar to track in the trochlear groove. Furthermore, our technique enables use of an additional external sensor for measurement of patellar tracking in all six degrees of freedom, which has been shown to deliver interesting results with regard to patellar tilt. However, both techniques seem to be superior to previously applied methods using fixations that completely prevented patellar tilt and rotation [9] or a ball joint anterior to the patella that introduced an additional torque [39].

Another reason for developing a completely new technique was our intention to create a testing rig that provides the possibility to undertake both knee (and patellar) kinematics and patellar stability measurements. Patellar maltracking and instability are strongly related from a clinical viewpoint, so it is considered a great advantage that they can both be analyzed experimentally using the same testing rig with the same knee specimens. In this regard, our experimental setting is a particular advancement for future investigation of surgical techniques for patellar stabilization (e.g. reconstruction of the medial patellofemoral ligament, trochleoplasty, osteotomy of the tibial tubercle), optimization of patellofemoral motion, and design of total knee arthroplasty. Furthermore, the combined data of our kinematics and stability measurements are an excellent basis for validation of a detailed knee model for computer simulation (e.g. finite element method or multibody dynamics), which can complement and partly substitute future experiments.

Another interesting finding of our study was the influence of asymmetric quadriceps loading on lateral patellar displacement and its dependence on the muscle force level. For small muscle forces, no relevant effect was visible. In this case, the influence of the passive stabilization structures predominates. For a well-balanced relationship between quadriceps muscle force and patellar displacement force, the effect of different load distributions was clearly visible and statistically significant. More lateral muscle loading yielded larger lateral shift and tilt, whereas more medial muscle loading yielded smaller lateral shift and medial tilt. Our results for more lateral muscle loading correspond to previous findings [24] with relaxed VMO. For higher muscle forces, the effect of asymmetric muscle loading disappeared again. This finding could be attributed to the very small displacements at these high muscle levels. The effect for mainly lateral muscle loading was more distinct than for mainly medial muscle loading and less sensitive to the force level. With regard to medial displacement, almost no effect of asymmetric muscle loading was visible. This observation again confirms the findings in [24] for missing VMO and even complements these previous findings by adding results for a weakened lateral quadriceps part. With respect to patellar tilt, our results for asymmetric muscle loading confirm our observations for different muscle force levels. Again, patellar shift and tilt were strongly related, and an effect on patellar shift also resulted in an effect on patellar tilt.

From a clinical viewpoint, the current results for asymmetric quadriceps loading are of particular interest with respect to patellar instability—especially in the lateral direction, where almost all patellar luxations occur. We could demonstrate that, in a range of medium quadriceps forces, asymmetric force distribution could significantly influence patellar mediolateral displacement and, thus, patellar stability. These results complement the findings of our previous kinematics studies, in which a significant effect of asymmetric muscle loading on patellar tilt [20] and the mediolateral shift of the center of patellofemoral pressure during simulated squatting motion [27, 31] was shown. In the present study, we could further demonstrate that, for higher quadriceps forces, almost no mediolateral patellar displacement was measured, independent of the force distribution. Thus, from a biomechanical viewpoint, selective training of the m. quadriceps in general and the m. vastus medialis in particular can be clearly recommended to improve patellar stability. However, the findings of biomechanical in vitro studies must be interpreted carefully and, for a specific patient, many more factors must be taken into account.

Although many advantages of our experimental setup (i.e. more physiologic, upright testing position, the minimally invasive force application, and the possibility for additional measurement of patellar tilt) have been shown, some limitations were present. The muscle forces were applied by servo motors and the force control of the motors was disturbed by the displacement force of the robot. Hence, the specified force levels could be reached only in the steady state at the end of each measurement. Therefore, the force–displacement curves as given in [9, 25] could not be measured with our setup. As mentioned above, our method of force application might influence patellar tilt, but this is unavoidable during such kind of measurements as long as the center of rotation of the patella cannot be tracked. Furthermore, we started our measurements in 15° of flexion since due to technical requirements of the servo motor control data could not be collected at 0°. This will be developed in future experiments. Another limitation could be the restriction to three quadriceps parts. However, the overall resultant force acting on the patella is important and, as shown above, the direction of the resultant force in our setting is comparable with similar systems in which five quadriceps parts were included and loadings were applied according to the PCSA. Finally, the combination of our complex industrial robot and the custom-made knee simulator is difficult to operate as well as being time- and cost-consuming in acquisition and application.

## Conclusion

A new experimental setup for reproducible measurement of patellar stability in an upright position under varying muscle loading was established. The setup enables a minimally invasive force application and unconstrained motion of the patella that can be recorded in all six degrees of freedom. Additional measurement of patellar tilt provided new insights into displacement mechanisms, which were shown to be different in medial and lateral directions. For lateral displacement, the patella tilted in the direction of force application (laterally), and tilted in the opposite direction (again laterally) for medial displacement. With regard to asymmetric quadriceps loading, we demonstrated that lateral patellar stability was influenced significantly. In particular increased force application on the vastus lateralis muscle led to a clear increase of lateral patellar displacement. Using adequate testing parameters, the developed experimental procedure was found to be an excellent method for the in vitro analysis of patellar stability, as well as for future investigation of surgical techniques for patellar stabilization and total knee arthroplasty.
